# Artificial oxygen carriers as a possible alternative to red cells in clinical practice

**DOI:** 10.1590/S1516-31802009000200008

**Published:** 2009-07-06

**Authors:** Fabiano Timbó Barbosa, Mário Jorge Jucá, Aldemar Araujo Castro, José Lira Duarte, Luciano Timbó Barbosa

**Affiliations:** 1 MSc. Professor in the Department of Clinical Surgery at Universidade Federal de Alagoas (UFAL), Maceió. Alagoas, Brazil.; 2 MD, PhD. Professor in the Department of Clinical Surgery at Universidade Federal de Alagoas (UFAL), Maceió. Alagoas, Brazil.; 3 MD, MSc. Professor in the Department of Public Health at Universidade Estadual de Ciências da Saúde de Alagoas, Maceió. Alagoas, Brazil.; 4 MD. Anesthesiologist at “Dr Armando Lages” Emergency Unit, Maceió, Alagoas, Brazil.; 5 MD. Clinician in the Department of Clinical Medicine at “Dr Armando Lages” Emergency Unit, Maceió. Alagoas, Brazil.

**Keywords:** Hemoglobins, Anemia, Blood, Blood transfusion, Blood substitutes., Hemoglobinas, Anemia, Sangue, Transfusão de sangue, Substitutos sanguíneos.

## Abstract

Fluid resuscitation is intended to eliminate microcirculatory disorders and restore adequate tissue oxygenation. The safety limits for a restrictive transfusion policy are given by patients’ individual tolerance of acute normovolemic anemia. Artificial oxygen carriers based on perfluorocarbon or hemoglobin are attractive alternatives to allogenic red blood cells. There are many risks involved in allogenic blood transfusions and they include transmission of infections, delayed postoperative wound healing, transfusion reactions, immunomodulation and cancer recurrence. Regardless of whether artificial oxygen carriers are available for routine clinical use, further studies are needed in order to show the safety and efficacy of these substances for clinical practice.

## RESUMO

Os fluidos de ressuscitação são utilizados para abolir os distúrbios microcirculatórios e restaurar a adequada oxigenação tissular. Os limites de segurança na prática da transfusão restrita são dados pela tolerância individual do paciente na anemia normovolêmica aguda. Os transportadores artificiais de oxigênio baseados no perfluorocarbono e na hemoglobina são opções atrativas para a transfusão alogênica de células vermelhas. Os riscos da transfusão sanguínea alogênica são muitos e incluem a transmissão de infecções, retardo na cicatrização pós-operatória, reações transfusionais, imunomodulação e risco de recorrência do câncer. Independentemente de os carreadores artificiais de oxigênio estarem disponíveis para o uso clínico rotineiro, mais estudos são necessários para mostrar a segurança e a eficácia dessas substâncias na prática clínica.

## INTRODUCTION

Blood transfusion is a universal procedure used in clinical and medical practice. Over recent years, the use of artificial oxygen carriers has been receiving much attention. The reasons for this have been the increasing cost of collecting and processing blood, public concerns about the safety of blood products, complications from blood transfusions, military requirements for increased volumes of blood during military conflicts and a fall in the number of new donors.^1^ Artificial oxygen carriers are synthetic solutions with the ability to bind, transport, and unload oxygen in the body.^2^ Some authors prefer the term oxygen therapeutics because of the multiple possible indications for these substances.^2^

In 1949, Amberson et al. reported the first infusion of cell-free hemoglobin in humans.^3^ They infused small volumes of hemoglobin solutions and reported a pressor response and resuscitation of a young woman with severe postpartum hemorrhagic shock.^3^ Perfluorocarbon (PFC) emulsions were introduced to the medical field in 1966, through an experiment using a mouse immersed in a liquid consisting of oxygenated PFC emulsions, in which the mouse survived.^3^

The ideal characteristics for blood substitutes are: nonantigenic properties; similarity to natural hemoglobin in terms of oxygen and carbon dioxide transport and delivery; absence of renal toxicity; stability at room temperature; sufficient half-life in circulation; long shelf life; ease of use; no overloading of the reticuloendothelial system; and low cost.^4^^,^^5^^,^^6^ The development of modified hemoglobin solutions in the 1980s was an improvement, in which the products showed stability and fewer side effects.^5^ Arterial oxygen carriers based on perfluorocarbons or hemoglobin-based oxygen carriers (HBOCs) are alternatives to allogenic red blood cells (RBCs).^7^

## NORMOVOLEMIC ANEMIA

It has been known for a long time that normal oxygen supply and tissue oxygenation do not depend on normal hemoglobin concentration if normovolemia is maintained.^8^^,^^9^ Initially, dilutional anemia is compensated by increased cardiac output, which at first is caused by an increase in left ventricular stroke volume.^7^ In deeper stages of normovolemic anemia, this is accompanied by an increase in heart rate.^7^ Oxygen delivery to the tissues (DO_2_) begins to decrease when hematocrit is lower than 25%.^7^ At this hematocrit level, DO_2_ starts to fall below the baseline level because the increased cardiac output becomes exhausted.^7^

The mechanisms involved in keeping up with tissue oxygenation demands include increased tissue oxygen extraction, such that the total body oxygen consumption (VO_2_) initially remains unchanged.^7^^,^^10^ At extreme degrees of dilutional anemia, the amount of oxygen delivered to the tissues becomes insufficient to meet their oxygen demand, and DO_2_ starts to decline.^11^

Myocardial performance is one variable that determines anemia tolerance, through coronary vasodilation and increased coronary blood flow.^7^ In patients with restricted coronary blood flow and limited ventricular performance who are using cardiodepressive medication, anemia tolerance is reduced.^12^

## ARTIFICIAL OXYGEN CARRIERS

Alternatives to allogenic RBCs have been researched in an effort to circumvent the adverse effects of transfusion.^6^ The risks involved in transfusions include transmission of infections, delayed postoperative wound healing, transfusion reactions, transfusion-related acute lung injury, immunomodulation and the potential for cancer recurrence.^13^^,^^14^^,^^15^ The incidence of transmission of the human immunodeficiency virus (HIV) by means of blood transfusions is around one in one million to one in 2.5 million units of blood, and the risk of hepatitis C is about one in 100,000 to one in 350,000 units.^4^ Noninfectious complications also exist with allogenic transfusions.^2^ Some examples are hemolytic transfusion reactions, transfusion-related lung disease, graft-versus-host disease, anaphylactic reactions and post-transfusion purpura.^2^ Certain techniques that are useful for reducing the need for allogenic blood transfusions during surgery have been developed. These include preoperative autologous blood donation, preoperative use of erythropoietin, acute normovolemic hemodilution, perioperative blood salvage, pharmacological treatment (antifibrinolytic drugs, desmopressin and anti-inflammatory agents), hypotensive anesthesia, surgical techniques, acceptance of minimal hemoglobin levels and blood substitutes.^2^^,^^6^ One attractive alternative to allogenic RBCs is synthetic blood substitutes, which can be applied independently of blood-group typing.^7^ Currently, research on blood substitutes is centering on synthetically manufactured perfluorocarbons and hemoglobin solutions, i.e. solutions based on human or bovine hemoglobin isolates.^2^^,^^6^^,^^7^^,^^16^

## PERFLUOROCARBON

PFCs are carbon-fluorine compounds characterized by high gas-dissolving capacity, low viscosity, and chemical and biological inertness.^17^ They are constructed molecules derived from cyclic or straight-chain hydrocarbons, with hydrogen atoms replaced by halogens with a molecular weight of 450 to 500 Daltons.^4^^,^^7^ PFCs are virtually immiscible with water and therefore have to be emulsified for intravenous application.^7^^,^^17^ A stable 60% emulsion has now been developed (58% perfluorooctyl bromide and 2% perfluorodecyl bromide) and thus a relatively highly concentrated emulsion that is clinically well tolerated now exists.^18^ PFCs have extremely small size (0.2 μm in diameter) and have the ability to enter the microcirculation.^4^

PFCs do not have the oxygen-bonding properties of hemoglobin but act as simple solvents, and the transportation and release of gases are based on physical solubility.^6^ Their oxygen kinetics are characterized by a linear relationship between partial pressure of arterial oxygen and oxygen content.^7^ The quantity of gas dissolved is linearly related to its partial pressure and, when the partial pressure of arterial oxygen (pO_2_) increases, the amount of oxygen carried by PFCs increases too.^4^^,^^6^ Oxygen release to the tissues is almost complete when there is a high pO_2_ gradient between arterial blood and the tissues.^7^ High pO_2_ is required to maximize the amount of oxygen transported.^7^ As PFCs can only dissolve oxygen and there is no binding function, sufficient oxygen carrying can only occur when the patient is breathing 70-100% oxygen.^6^

At the present time, the metabolism of PFCs in humans is not completely known.^17^ The PFC emulsion droplets are rapidly taken up by the reticuloendothelial system (RES).^6^^,^^7^^,^^17^ This uptake into the RES determines the intravascular half-life.^19^ In the RES, the emulsion droplets are slowly broken down and taken up by the blood again. The PFCs are then transported to the lungs, where any unaltered molecules are excreted via exhalation.^17^ To avoid RES overload and consequent immunosuppression, the clinical application of PFC solutions is restricted to low dosages.^4^^,^^7^

PFC emulsions have side effects. The adverse effects include: flu-like symptoms and complementary and phagocytic activation.^6^ Flu-like symptoms are an acute effect involving transient facial flushing, backache (particularly during the infusion period) and fever.^6^^,^^17^^,^^20^ Bronchospasm and thrombocytopenia, secondary to RES activation, have been demonstrated.^2^ Transient thrombocytopenia generally begins three to four days after PFC administration, reaches an average of 30% to 40% and returns to normal after seven to ten days.^2^

The advantages of PFCs are their low cost, long shelf life (two years), lack of vasopressive effects, small size and synthetic nature (without the possibility of pathogen transfusion).^4^ The disadvantages are the flu-like symptoms, need for high oxygen concentration, rapid plasma clearance and low capacity for carrying oxygen at physiological pO_2_ levels.^4^

## HEMOGLOBIN-BASED OXYGEN CARRIERS

Hemoglobin is an obvious candidate for a blood substitute, with a number of desirable characteristics.^21^ It has a high capacity to carry oxygen; it lacks the numerous and complex antigens of the RBC membrane, and hence it is universally compatible; and it is a robust molecule that withstands rigorous purification and viral inactivation processes.^21^ The hemoglobin used for manufacturing HBOCs originates from out-of-date human red cells or from bovine blood, or is genetically engineered.^2^^,^^5^^,^^7^^,^^17^

Once the hemoglobin moiety has been removed from the protective environment of the RBC membrane, low concentrations of 2,3-diphosphoglycerate (2,3-DPG) are present. These cause the oxyhemoglobin dissociation curve to left-shift (decrease in P_50_). The hemoglobin tetramers dissociate into their component ab dimers, which undergo rapid elimination by the kidneys, from which renal toxicity may result.^5^ Purified hemoglobin molecules are chemically modified to increase their stability and to modulate oxygen affinity.^6^ These chemical modifications include intramolecular cross-linking, polymerization using glutaraldehyde or o-raffinose, conjugation of polyethylene glycol, insertion of 2,3-DPG analogues or embedding of hemoglobin molecules into phospholipid vesicles.^7^ Based on techniques for improving molecular stability, four groups of hemoglobin solution are currently available: surface-modified hemoglobin, intramolecular cross-linked hemoglobin, polymerized hemoglobin and liposome-encapsulated hemoglobin.^5^ These products do not have antigenic features and do not require compatibility testing.^4^

The HBOCs under development all have vascular half-lives in the range of 18 to 24 hours, which is adequate for most acute care applications.^21^ Most can be stored at 4 ˚C or at room temperature for 1-2 years and none of them require any form of compatibility testing.^21^

The oxygen affinity of most HBOCs is lower than that of native human blood, thus facilitating the offloading of oxygen to the tissues, as indicated by high P_50_ values.^7^^,^^22^ Bovine hemoglobin does not have 2,3-DPG and its P_50_ remains in the range of 30 mmHg, whether in or out of the RBCs.^5^

Extracellular hemoglobin possesses strong vasoconstrictive properties. The underlying mechanisms explaining this action are the scavenging of nitric oxide, augmented release of endothelin and stimulation of endothelin receptors and adrenoceptors.^7^

Cell-free hemoglobin has oncotic properties and increases the blood volume by an amount greater than the transfused volume.^5^^,^^23^ This action may be beneficial when plasma expansion is required in cases of shock resuscitation but can also be harmful in the absence of hypovolemia.^5^ Because of these oncotic properties, HBOCs may be used as fluid resuscitation in cases of hemorrhagic shock, and for treating surgical blood loss.^7^

Hemoglobin solutions have many adverse effects. Their vasoactivity increases the systemic and pulmonary arterial pressure and increases the systemic and pulmonary vascular resistence.^5^ Stroma-free hemoglobin results in oliguria secondary to acute tubular necrosis.^5^ Cardiac output may decrease following infusion.^8^ Clinical findings of abdominal discomfort, pain, nausea, vomiting and increased enzyme activity have been reported.^5^^,^^7^ Free hemoglobin increases platelet aggregation by means of nitric oxide scavenging.^5^

To achieve effective reduction of RBC transfusions, HBOCs must be infused over a long period, theoretically until erythropoiesis can provide autologous RBCs.^7^

The advantages of HBOCs are that they increase the oxygen content of blood; have a good capacity for carrying oxygen at physiological pO_2_ levels; are free from infection risk; have a small size; and do not need typing and cross-matching to achieve compatibility.^4^

The disadvantages of HBOCs are that their use can cause hypertension, renal toxic effects, sepsis, cytotoxic effects and esophageal dysfunction.^4^

## FINAL CONSIDERATIONS

Although the incidence of transfusion-transmitted HIV, hepatitis B virus and hepatitis C virus has been greatly reduced since the 1980s, the threat of new or emerging pathogens, such as Creutzfeldt-Jakob disease (including bovine spongiform encephalopathy), hepatitis G virus, human T-cell leukemia virus, bacterial contamination and corona virus (responsible for severe acute respiratory syndrome), continue to motivate research on oxygen carriers.^2^^,^^22^

In elective surgery, in which substantial blood loss is anticipated, PFCs can be used.^7^ The combination of hyperoxic ventilation and repeated co-administration of small boluses of PFCs maintains adequate tissue oxygenation.^7^ A higher rate of neurological complications has been found in human cardiac surgery cases.^24^

The first randomized controlled trial using human polymerized hemoglobin in cases of acute blood loss was conducted in 1998. The results showed that the use of allogenic blood cells was lower. There were no serious or unexpected adverse events in the experimental group.^25^

One single-blind randomized study used perflubron emulsion prior to non-cardiac surgery and demonstrated that the transfusion requirements during high-blood-loss surgery could be lower than in the control group.^26^ The incidence of adverse events in the two groups was similar, but greater numbers of such events were reported in the PFC group for the cardiovascular and digestive systems.^26^

In experimental studies on severe shock, resuscitation using HBOCs consistently stabilized the hemodynamics and tissue oxygenation.^27^ The potential uses for HBOCs in the future include cases of shock, organ ischemia, red blood cell incompatibility, acute lung injury, transplant organ preservation, sickle cell anemia, tumor therapy and air embolism.^6^

The potential advantages offered by oxygen carriers include universal compatibility, one to three years of shelf life compared with 42 days for blood cells, the ability to be produced in large quantities, a manufacturing process that can reduce the risk of infectious agents, reduced dependence on donor blood supplies and an alternative for patients who will not accept transfusions of red blood cells, such as Jehovah’s Witnesses.^28^

HBOCs and PFCs have so far not been licensed by the National Agency for Sanitary Surveillance (Agência Nacional de Vigilância Sanitária) for use in Brazil. In the United States, the Food and Drug Administration has discontinued perflubron use. A systematic review is being conducted by the Cochrane Collaboration to examine the evidence regarding the safety and efficacy of methods for avoiding red blood cell transfusion.^1^

## CONCLUSION

The risks associated with blood transfusions have been well described in the literature. Artificial oxygen carriers are a step closer to being clinically used in humans. Regardless of whether artificial oxygen carriers are available for routine clinical use, further studies are needed in order to show the safety and efficacy of these substances for clinical practice.
